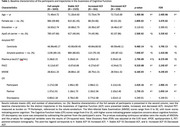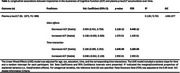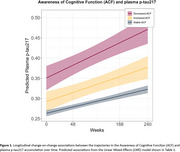# Distinct awareness trajectories and plasma *p*‐tau217 accumulation over time in preclinical Alzheimer's disease

**DOI:** 10.1002/alz70857_101104

**Published:** 2025-12-24

**Authors:** David López‐Martos, Ibai Diez, Oriol Grau‐Rivera, Gonzalo Sánchez‐Benavides, Patrizia Vannini

**Affiliations:** ^1^ Barcelonaβeta Brain Research Center (BBRC), Pasqual Maragall Foundation, Barcelona, Spain; ^2^ Massachusetts General Hospital, Harvard Medical School, Boston, MA, USA; ^3^ Hospital del Mar Research Institute (IMIM), Barcelona, Spain; ^4^ Athinoula A. Martinos Center for Biomedical Imaging, Charlestown, MA, USA; ^5^ Gordon Center for Medical Imaging, Massachusetts General Hospital, Boston, MA, USA; ^6^ Ikerbasque, the Basque Foundation for Science, Bilbao, Bizkaia, Spain; ^7^ Biobizkaia HRI, Barakaldo, Bizkaia, Spain; ^8^ Servei de Neurologia, Hospital del Mar, Barcelona, Spain

## Abstract

**Background:**

Increased and decreased Awareness of Cognitive Function (ACF) may both represent early symptoms in the preclinical stage of Alzheimer's disease (AD). The underlying pathology of these two states remains unclear, with the latter being particularly important as loss of insight (anosognosia) is a devastating symptom that affects up to 80% of patients in the AD dementia stage. Specifically, understanding the ACF relationship with AD pathophysiology could provide key missing insights to predict the onset of anosognosia. Here, we identified distinct trajectories of ACF over 4.5 years in individuals who were cognitively unimpaired (CU) at baseline and further examined their cross‐sectional association with amyloid PET and longitudinal association with plasma *p*‐tau217.

**Method:**

We analyzed 1,643 CU individuals who were part of the A4 and LEARN cohorts. The standardized participant‐partner discrepancy of the Cognitive Function Index was regressed in a Linear Mixed‐Effects (LME) model to define 3 groups based on their longitudinal regression slopes: individuals demonstrating increased, stable, and decreased ACF trajectories over time. ANOVAs were used to assess baseline differences. LME models were used to examine the change‐on‐change relationship between distinct ACF trajectories and plasma *p*‐tau217 accumulation over a 4.5‐year period.

**Result:**

The decreased ACF trajectory had higher baseline amyloid burden (*p*‐value=9.48E‐14) as compared to both stable and increased ACF trajectories, but no difference was observed for the increased trajectory as compared to the stable ACF trajectory (Table 1). The decreased ACF trajectory showed a main effect of higher plasma *p*‐tau217 (Std. Coefficient=0.594; 95%CI=0.431, 0.756) and an interaction with time indicating higher accumulation of plasma *p*‐tau217 (Std. Coefficient=0.166; 95%CI=0.080, 0.253), as compared to the stable ACF trajectory. In contrast, no main/interaction effect was observed for the increased ACF trajectory as compared to the stable trajectory (Table 2, Figure 1).

**Conclusion:**

The decreased ACF trajectory strongly aligned with AD pathology, highlighting its role as a potential marker of the disease. The integration of a non‐invasive plasma biomarker with awareness represents a groundbreaking approach, providing novel insights into the earliest changes of awareness in AD. This combined clinical‐biological strategy could pave the way for earlier detection strategies and improved understanding of preclinical disease dynamics.